# Feasibility and age-related trends of a home-based online exercise program in adolescents with idiopathic scoliosis

**DOI:** 10.3389/fped.2025.1695963

**Published:** 2025-12-16

**Authors:** Jing Wang, Youcun Su

**Affiliations:** 1 School of Sports Science and Engineering, East China University of Science and Technology, Shanghai, China; 2 College of Physical Education and Health, East China Normal University, Shanghai, China

**Keywords:** idiopathic scoliosis, Schroth, Pilates, online rehabilitation, adolescents

## Abstract

**Background:**

Idiopathic scoliosis (IS) is a three-dimensional spinal deformity that often progresses during adolescence. While bracing and exercise therapies are standard conservative treatments, limited research has examined the feasibility of fully online, home-based combined exercise programs and the clinical trends observed during participation —especially in adolescents undergoing brace treatment. This single-arm prospective cohort study (without a control group) aimed to evaluate the feasibility of a six-month, fully online, home-based Schroth-Pilates program combined with brace treatment in adolescents with IS, and to observe potential age-related trends in clinical measures and adherence.

**Methods:**

A single-center prospective cohort design was used involving 114 adolescents with IS (Cobb angle 10°–45°) receiving standard brace treatment. Participants engaged in supervised online Schroth-Pilates sessions three times per week for six months. Primary measures included Cobb angle and ATR, recorded at baseline and post-program. Feasibility was assessed via program completion and adherence rates. Mixed-design ANOVAs were used to explore trends over time and between age groups (10–13 vs. 14–17 years).

**Results:**

The overall program completion rate was 97.4%, with no significant adherence differences between age groups. Significant improvements were observed in Cobb angle [F(1,112) = 16.42, *p* < .001, *η*^2^ = .255] and ATR [F(1,112) = 11.87, *p* = .001, *η*^2^ = .198], both exceeding the minimum clinically important difference for adolescents with IS. Higher adherence was positively correlated with greater reductions in Cobb angle and ATR, suggesting that consistent participation enhanced treatment outcomes. Younger participants demonstrated greater mean improvements, possibly reflecting higher flexibility and growth potential.

**Conclusion:**

A fully online, home-based Schroth–Pilates program is feasible and clinically effective for adolescents with IS, achieving high adherence and meaningful improvements in spinal curvature and trunk rotation—particularly among younger participants. These results support the clinical viability of virtual scoliosis rehabilitation and highlight the need for future controlled and longitudinal studies to confirm long-term outcomes.

## 1 Introduction

IS is a complex three-dimensional spinal deformity that typically emerges during periods of rapid growth, most commonly in adolescence. It is primarily characterized by a lateral curvature in the coronal plane, often accompanied by rotational deformities and, in some cases, alterations in the sagittal alignment (1). Several etiological factors may influence its progression, including age at onset, sex, ventral spinal overgrowth, severity of initial curvature, and functional tethering of the spinal cord (2). As the condition advances, it can lead to postural misalignment, muscular imbalance, limited pelvic mobility, and gait alterations—factors that contribute to functional limitations, chronic low back pain, and potential social or psychological burdens (3).

Balance impairments in IS are often attributed to increased trunk rotation and asymmetrical loading of the spine (4). Additionally, kinematic changes in lower limb joints, altered foot pressure distribution, reduced cadence, increased gait speed, and shortened step length have been observed (5, 6). These biomechanical adaptations increase energy expenditure and may negatively affect aerobic capacity, neuromuscular control, and sensory integration (7).

Considering that physical abnormalities originating in childhood or adolescence often persist into adulthood if left untreated, there is a pressing need for timely diagnostic and therapeutic strategies to prevent long-term consequences. Currently, conservative treatment is generally favored over surgical intervention in adolescents with IS, with evidence suggesting that such approaches can reduce the rate of surgery by up to 50% (8, 9).

Among conservative strategies, spinal bracing is a cornerstone intervention, commonly prescribed to prevent curve progression until skeletal maturity. Some studies have shown that brace use can reduce the worsening of scoliosis by 50% to over 80%, significantly decreasing the need for surgical correction (10, 11).

Exercise-based therapies also play a vital role in IS management. The Schroth method, a scoliosis-specific exercise system, emphasizes individualized, three-dimensional corrective movements designed to de-rotate, elongate, and stabilize the spine (12, 13). Pilates, another non-invasive method, focuses on postural alignment, core strength, and breathing coordination. It has been associated with improvements in flexibility, physical and mental well-being, and even Cobb angle reduction in individuals with IS (14, 15). Some studies suggest that Pilates can contribute to scoliosis management by strengthening postural muscles and improving body balance (16).

The effectiveness of exercise interventions is often related to their duration, with programs exceeding six months showing greater improvements than shorter ones (17). However, prolonged rehabilitation protocols may compromise adherence, particularly in adolescent populations (18). Therefore, combining two complementary exercise methods—such as Schroth and Pilates— may support long-term engagement and address multiple aspects of spinal health, yet this approach has received limited attention in scoliosis research, especially in youth populations undergoing brace treatment.

Age has been identified as a potential factor influencing the response to conservative scoliosis treatments. Younger adolescents often present with more flexible spines, greater remaining growth potential, and potentially better responsiveness to brace therapy and corrective exercises modulation (19). Furthermore, differences in cognitive development, adherence capacity, and parental involvement may affect engagement and outcomes across age groups. Yet, few studies have examined whether treatment effects differ between younger and older adolescents participating in structured exercise programs.

Recent studies have emphasized the need for innovative strategies to enhance adherence to conservative treatment and to understand neurophysiological and balance mechanisms in AIS (5, 20). In recent years, there has been growing interest in delivering scoliosis-specific exercise programs through online platforms, especially to overcome geographic barriers, increase accessibility, and support long-term adherence. Tele-rehabilitation offers advantages including improved accessibility, reduced travel burden, and potential to maintain treatment continuity during public-health restrictions. However, tele-rehabilitation modalities may face limitations such as reduced tactile feedback, variable home environments, and reliance on patient/caregiver technology literacy—factors that can influence fidelity and outcomes (21). These considerations motivated the present investigation of a fully online supervised Schroth–Pilates program delivered alongside brace treatment. Therefore, this prospective cohort study aimed to evaluate the feasibility of a fully online home-based Schroth-Pilates program in adolescents with IS undergoing brace treatment and to observe age-related trends in adherence and clinical measures over a six-month monitoring period.

## 2 Materials and methods

### 2.1 Study design and participants

This prospective cohort study was conducted to evaluate the feasibility of a fully online, home-based Schroth-Pilates exercise program combined with brace treatment and to observe age-related trends in adherence and clinical measures over a six-month monitoring period. Participants were recruited in June 2024, and data collection was carried out from July to December 2024 in Shanxi Province, China. The exercise program and brace treatment were part of the participants' standard care plan, prescribed by their treating orthopedic specialists. The research team's role was limited to monitoring adherence, collecting clinical data, and evaluating program feasibility.

Inclusion criteria were adolescents aged 10–17 years, consistent with the International Scientific Society on Scoliosis Orthopedic and Rehabilitation Treatment (SOSORT) guidelines (22). Additional criteria included: a diagnosis of IS with a Cobb angle between 10° and 45°, and ATR ≥7°, a prescription for brace treatment, the ability to participate in a six-month online exercise program, and access to a stable internet connection and suitable digital device. Exclusion criteria included non-IS, planned or prior spinal surgery, contraindications to exercise, intellectual disability, autism spectrum disorders, or diagnosed neurological/rheumatic conditions.

A power analysis using G*Power 3.0.10 indicated a minimum sample size of 65 participants to detect a moderate effect size (*f* = 0.40) with 80% power and *α* = 0.05, based on Cohen's guidelines (23). To account for a potential dropout rate of 15%, the recruitment target was set at 75 participants.

This study protocol was reviewed and approved by the Ethical Committee of East China University of Science and Technology, China (2024ECUST0202). Also, it was conducted in accordance with the latest Declaration of Helsinki. Written informed consent was obtained from all participants' parents or legal guardians prior to enrollment. This study was reported following the STROBE checklist for cohort studies (Strengthening the Reporting of Observational Studies in Epidemiology). The completed STROBE checklist is provided as Supplementary Material S1.

### 2.2 Measurements

Measurements were conducted at baseline and after six months. All assessments took place at the same orthopedic clinic in Shanxi Province by trained personnel who were blinded to age group classification.

Participants' height and weight were measured using standardized equipment, and body mass index (BMI) was calculated as weight in kilograms divided by height in meters squared (kg/m^2^).

The Cobb angle was assessed using standard radiographic procedures. Standing anteroposterior full- radiographs were obtained, and the most tilted vertebrae at the top and bottom of each curve were identified. The angle between the superior endplate of the upper vertebra and the inferior endplate of the lower vertebra was measured using Surgimap software (version 2.3.2.1; Nemaris, USA), a validated digital tool for spinal analysis (24). All measurements followed the SOSORT imaging guidelines and were performed by an experienced orthopedic specialist blinded to the study timeline.

ATR was measured using a scoliometer (Pedi-Scoliometer, Pedihealth, Finland), following a protocol similar to the Adam's forward bend test. Participants stood upright with feet together and bent forward until their back was parallel to the floor. The scoliometer was placed along the spine at the point of maximal rib prominence, and the highest ATR value was documented (25).

To ensure measurement reliability, intra- and inter-rater reliability for Cobb angle and ATR were assessed in a random subsample of 12 participants. The intra-rater ICC (ICC [3,1]) for Cobb angle was 0.92 (95% CI: 0.84–0.95) and the inter-rater ICC (ICC [2,1]) was 0.90 (95% CI: 0.81–0.93). All reliability analyses were performed using SPSS v26. These values indicate excellent measurement consistency.

Feasibility was assessed using two indicators: (1) adherence rate, calculated as mean number of completed exercise sessions out of the total scheduled sessions (72 sessions across 6 months), and (2) program completion rate, defined as the percentage of participants who completed the entire six-month protocol. Exercise adherence was verified through three sources: (1) therapist attendance records, (2) platform login timestamps, and (3) participant weekly exercise logs. Cross-validation between therapist logs and platform data showed 98% agreement, confirming high accuracy of adherence records.

### 2.3 Home-based online exercise program

All participants followed a six-month, fully online, home-based Schroth-Pilates program as part of their prescribed conservative treatment. Sessions were conducted three times per week, lasting 45–60 min, via a secure video conferencing platform (e.g., Zoom). All sessions were conducted by two certified physiotherapists who completed advanced Schroth and Pilates training (each >100 h). Both followed a standardized online checklist and participated in weekly fidelity meetings to ensure uniform supervision quality. Each session was equally divided between Schroth and Pilates exercises.

The Schroth component focused on three-dimensional spinal correction with rotational angular breathing and targeted postural control (25). The Pilates component emphasized core stability and neuromuscular coordination (26). Exercises were adapted for home settings using simple equipment (exercise balls, foam blocks, resistance bands). Across both modalities, participants performed 2–4 sets per exercise, with repetitions lasting 15–45 s or 8–25 counts, and rest intervals of 30–60 s. Progression and intensity were adjusted based on individual performance and tolerance. Certified physiotherapists supervised sessions remotely to ensure correct performance. Parents were encouraged to support younger participants to maintain adherence. Weekly check-ins provided additional monitoring and feedback. To support adherence, parents were encouraged to assist younger participants and help maintain a consistent home exercise schedule.

### 2.4 Brace treatment

All participants were prescribed and fitted with a standardized rigid thoracolumbosacral orthosis (TLSO), fabricated using computer-aided design/computer-aided manufacturing (CAD/CAM) technology. This approach ensured uniformity in brace design and fit across the sample. The CAD/CAM braces were constructed by certified orthotists following SOSORT recommendations, using either 3D scanning or computer-generated modeling based on individual spinal morphology (22). Each brace was custom-designed to provide three-point corrective pressure tailored to the participant's spinal curvature, flexibility, and growth potential.

Bracing was prescribed in accordance with SRS and SOSORT guidelines for conservative scoliosis management (22). Participants with Cobb angles between 20° and 40° were indicated for bracing due to curve magnitude and documented risk of progression, while those with 10°–19° were braced if other clinical indicators (family history, Risser <2, or rapid growth) suggested high progression risk (22). The primary goal of bracing was to stabilize or improve curve magnitude during growth and to prevent progression beyond the surgical threshold.

Brace fitting was performed in collaboration with orthopedic specialists at the study site. The prescribed wearing time was 15–18 h per day, including nighttime use, consistent with SOSORT and SRS recommendations advocating ≥16 h/day compliance for optimal curve stabilization. Brace-wearing duration remained unchanged throughout the study period. To objectively monitor brace adherence, all TLSOs were equipped with embedded Thermochron iButtons (Maxim Integrated Products, USA) that recorded internal temperature at 10-minute intervals. Data were collected monthly and used to calculate mean daily wear time. Participants and caregivers were blinded to the recording schedule to minimize behavioral bias. The bracing protocol was maintained as part of each participant's standard care while they concurrently engaged in the supervised online Schroth–Pilates program. No adjustments to brace design or wearing duration were made during the exercise intervention period.

### 2.5 Statistical methods

All statistical analyses were performed using SPSS version 26 (IBM Corp., Armonk, NY, USA), with a significance level set at *p* < 0.05. Descriptive statistics (means, standard deviations, and frequencies) were used to summarize participant characteristics, including age, sex, and baseline clinical measures. To observe baseline comparability between the two age subgroups (younger adolescents vs. older adolescents), independent-sample *t*-tests were conducted for continuous variables (e.g., Cobb angle, ATR, BMI), and chi-square tests were used for categorical variables (e.g., sex, family history of scoliosis).

To observe age-related trends in clinical measures (Cobb angle and ATR), a mixed-design ANOVA was performed with time (baseline vs. follow-up) as the within-subject factor and age group (10–13 vs. 14–17 years) as the between-subject factor. All relevant assumptions for mixed ANOVA—including normality, sphericity (for within-subject effects), and homogeneity of variance—were tested and met. In cases of significant main or interaction effects, Bonferroni-adjusted *post hoc* comparisons were performed. Effect sizes were reported using partial eta squared (*η*^2^_p_), with thresholds of 0.01 (small), 0.06 (moderate), and 0.14 (large) in accordance with established guidelines (23).

To investigate the influence of adherence on treatment outcomes, secondary analyses were conducted. Pearson correlation coefficients (*r*) were computed to assess the association between the number of completed exercise sessions and absolute changes in Cobb angle and ATR.

## 3 Results

**Figure 1:**
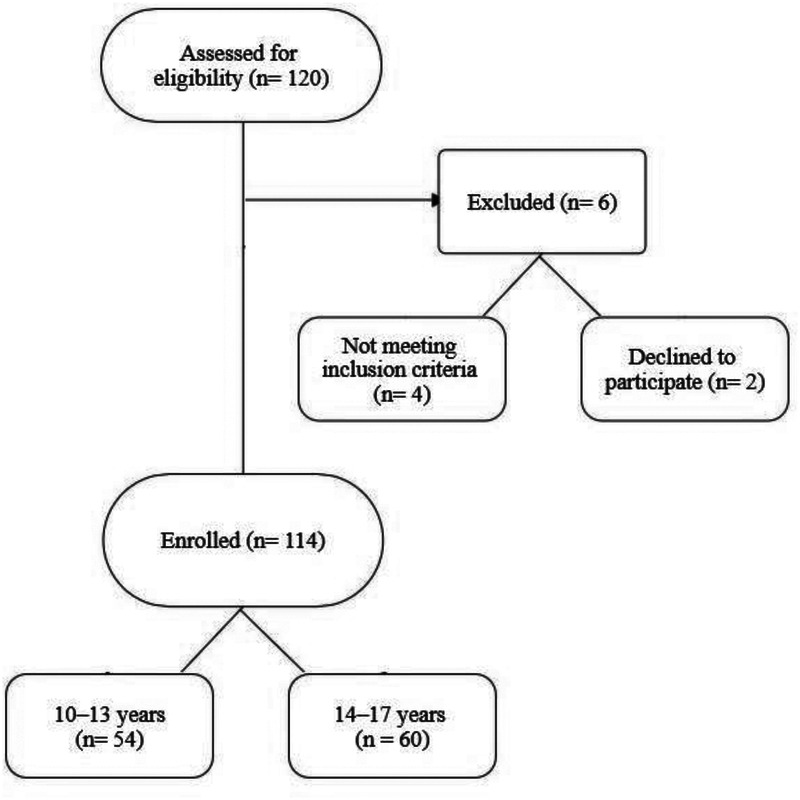
Participant flow diagram.

Out of 120 adolescents initially assessed for eligibility, 114 participants (mean age: 13.52 ± 1.10 years) met the inclusion criteria and were enrolled in the study (Figure 1). Of these, 54.38% were female, and 61.4% reported no family history of scoliosis. Participants were stratified into two age-based subgroups: younger adolescents (*n* = 54; aged 10–13 years) and older adolescents (*n* = 60; aged 14–17 years).

At baseline, the mean Cobb angle of the primary spinal curvature was 34.22 ± 2.95°, and the sum of all measured Cobb angles was 53.47 ± 12.15°. The maximum ATR was 12.88 ± 2.25° and BMI was 18.93 ± 2.10 kg/m^2^. Baseline demographic and clinical characteristics are summarized in Table 1. Independent-sample *t*-tests and chi-square tests showed that, apart from chronological age (*p* < 0.001, reflecting the intended age stratification), the two subgroups (10–13 years vs. 14–17 years) were largely comparable across all baseline variables. A small but statistically significant difference was noted in BMI (*p* = 0.042), with older adolescents exhibiting slightly higher mean values; however, this difference was not considered clinically meaningful. No other between-group differences were found for spinal measures (Cobb angle, sum of curves, or ATR) or family-history distribution (*p* > 0.05).

**Table 1:** Baseline characteristics of participants by age group (10–13 years vs. 14–17 years).

Variable	Total (*N* = 114)	Groups		*p*
		10–13 years (*n* = 54)	14–17 years (*n* = 60)	
Age (years), mean ± SD	13.52 ± 1.10	11.82 ± 0.89	15.12 ± 0.86	<0.001
Female participants, *n* (%)	62 (54.38%)	28 (51.85%)	34 (56.67%)	0.108
No family history of scoliosis, *n* (%)	70 (61.4%)	33 (61.11%)	37 (61.67%)	0.219
Cobb angle (°), mean ± SD	34.22 ± 2.95	33.87 ± 2.88	34.53 ± 3.01	0.194
Sum of curves (°), mean ± SD	53.47 ± 12.15	51.92 ± 11.87	54.88 ± 12.34	0.085
Maximum ATR (°), mean ± SD	12.88 ± 2.25	12.65 ± 2.12	13.09 ± 2.36	0.392
BMI (kg/m^2^), mean ± SD	18.93 ± 2.10	18.47 ± 2.05	19.34 ± 2.12	0.042

To observe changes in clinical measures and explore age-related trends during participation in the fully online, home-based Schroth-Pilates program, mixed-design ANOVAs were performed for Cobb angle and ATR (Table 2).

**Table 2:** Pre- and post-program comparison of cobb angle and ATR by age group.

Outcome variable	Group	Pre (Mean ± SD)	Post (Mean ± SD)	Main effect of time	Main effect of age group	Time × age group interaction
Cobb Angle (°)	10–13 years	33.39 ± 2.09	28.89 ± 1.78	F(1, 112) = 16.42, *p* < .001, *η*^2^ = .255	F(1, 112) = 5.03, *p* = .029, *η*^2^ = .095	F(1, 112) = 6.17, *p* = .016, *η*^2^ = .114
	14–17 years	34.11 ± 2.34	30.25 ± 2.12			
ATR (°)	10–13 years	12.91 ± 1.22	7.58 ± 1.09	F(1, 112) = 11.87, *p* = .001, *η*^2^ = .198	F(1, 112) = 4.21, *p* = .045, *η*^2^ = .081	F(1, 112) = 5.48, *p* = .023, *η*^2^ = .103
	14–17 years	13.13 ± 1.08	8.19 ± 0.98			

Cobb angle analysis revealed a significant main effect of time [F(1, 112) = 16.42, *p* < .001, partial *η*^2^ = .255], indicating a reduction over six months. Younger adolescents improved from 33.39 ± 2.09° 28.89 ± 1.78°, while the older adolescents improved from 34.11 ± 2.34° to 30.25 ± 2.12° (*p* < 0.001 for both). A significant main effect of age group was also observed [F(1, 112) = 5.03, *p* = .029, partial *η*^2^ = .095], along with a significant time × age group interaction [F(1, 112) = 6.17, *p* = .016, partial *η*^2^ = .114], suggesting that younger adolescents exhibited greater improvement in Cobb angle reduction.

For ATR, a significant main effect of time was found [F(1, 112) = 11.87, *p* = .001, partial *η*^2^ = .198]. The ATR decreased from 12.91 ± 1.22° to 7.58 ± 1.09° in the younger group, and from 13.13 ± 1.08° to 8.19 ± 0.98° in the older group (*p* < .001 for both). A significant main effect of age group [F(1, 112) = 4.21, *p* = .045, partial *η*^2^ = .081], and a significant interaction between time and age group [F(1, 112) = 5.48, *p* = .023, partial *η*^2^ = .103], further supported the observation that younger participants experienced more substantial improvements.

Significant main effects of age group and time × age group interactions suggested that younger participants exhibited greater observed changes.

**Figure 2:**
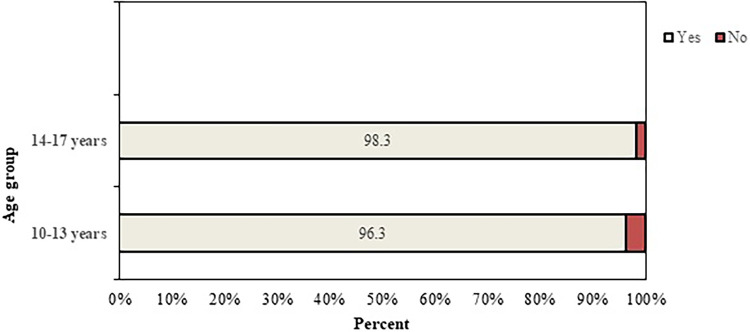
Program completion rate by age group; “Yes” indicates that the participant completed the program; “No” indicates that the participant did not complete the program.

The overall program completion rate was 97.4%, with 96.3% (52 out of 54) in the younger group and 98.3% (59 out of 60) in the older group (Figure 2). The difference between groups was not statistically significant [*χ*^2^(1) = 0.38, *p* = .54].

**Figure 3:**
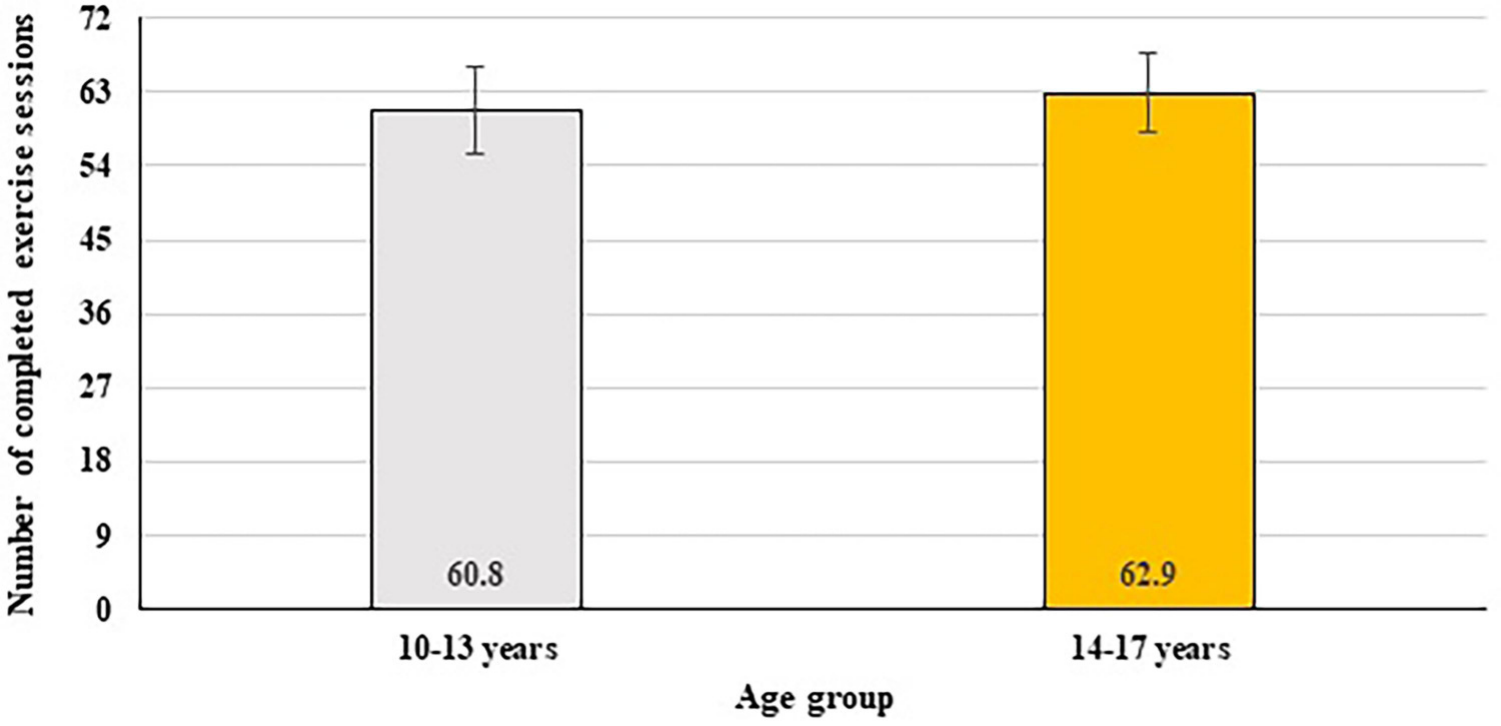
Comparison of the number of completed exercise sessions between younger and older adolescents.

An independent-samples t-test was conducted to compare the mean number of completed exercise sessions between the younger and older adolescents. The younger group completed an average of 60.8 ± 5.2 sessions, while the older group completed 62.9 ± 4.7 sessions out of 72. Although the older adolescents showed slightly higher adherence, the difference did not reach statistical significance [*t*(112) = –1.16, *p* = 0.109, Cohen's *d* = 0.11; Figure 3].

Correlation analyses revealed significant positive associations between adherence and clinical improvements. The number of completed sessions correlated with absolute change in ATR (*r* = 0.42, *p* = 0.001) and Cobb angle (*r* = 0.36, *p* = 0.004), indicating that participants who attended more sessions achieved greater rotational and angular correction.

## 4 Discussion

This prospective cohort study monitored adolescents with IS who participated in a six-month, fully online, home-based Schroth-Pilates program while receiving standard brace treatment as part of their standard care. Observable improvements in Cobb angle and ATR were noted across the study period, with younger participants (10–13 years) showing comparatively greater changes. These findings highlight the feasibility of integrating structured, remotely delivered exercise programs into conservative scoliosis management and provide insight into potential age-related trends in response.

The mean improvement of 5.39° in Cobb angle (15.87%) exceeds the commonly referenced clinical threshold of 5° for Cobb angle, which is often used as the minimum clinically meaningful change in IS (27). Supervised Schroth programs delivered in-person have shown modest but clinically relevant reductions in Cobb angle, with some randomized or controlled studies reporting average improvements of 2–4°, supporting the efficacy of structured scoliosis-specific exercise when supervised (27, 28). Similarly, studies examining Pilates as a single modality have generally reported smaller and more heterogeneous effects on spinal curvature and posture, particularly when programs were unsupervised or adherence was low (29). The combined Schroth–Pilates model used in the present study may have provided complementary biomechanical and neuromuscular benefits, integrating Schroth's three-dimensional correction with Pilates' emphasis on core stability and body awareness (14, 30).

When comparing our results with prior in-person interventions, several notable similarities and differences emerge. Although previous studies demonstrated benefits of supervised scoliosis-specific exercises on both Cobb angle and trunk rotation (27–29), our observed Cobb improvement is numerically larger than some reported in-person effects. This may reflect the dual-modality design (Schroth + Pilates), the higher session frequency and intensity, and the exceptionally high adherence observed in our cohort. Nevertheless, in-person programs offer tactile feedback and manual corrections that are difficult to replicate remotely—limitations that may contribute to inter-individual variability and should be acknowledged when interpreting effect sizes.

ATR outcomes in our cohort (mean reduction = 5.94°) are particularly noteworthy because rotational deformity is typically more resistant to conservative interventions than coronal curvature (31). Randomized and controlled Schroth-based trials have reported significant but generally smaller ATR reductions (and variable effect sizes) (28, 32), whereas combined Schroth–Pilates interventions and supervised programs have tended to show larger improvements programs (12, 33). These findings suggest that supervision, exercise specificity, and intervention dose are key determinants of rotational correction.

In addition to coronal and rotational improvements, future investigations should assess secondary or hidden asymmetries—such as leg-length discrepancy and breast or chest wall asymmetry—which can contribute to global postural imbalance and cosmetic concerns in adolescents with IS. Understanding the effect of exercise-based interventions on these secondary outcomes could better inform comprehensive rehabilitation strategies and patient-centred endpoints.

Another critical outcome was the high adherence rate (>97% completion and >84% session attendance), despite the fully online delivery. This indicates the acceptability and practicality of virtual exercise delivery, especially when sessions are supervised and individualized feedback is provided (34). Adherence is a recognized mediator of treatment efficacy in AIS, and our correlation analyses confirmed a significant positive association between adherence and both ATR (*r* = 0.42, *p* = 0.001) and Cobb angle improvements (*r* = 0.36, *p* = 0.004). This finding aligns with recent tele-rehabilitation studies showing that structured online programs with real-time supervision and caregiver involvement can maintain high attendance and clinical effectiveness (35, 36). Hence, the consistently high adherence in this cohort likely contributed to the relatively large magnitude of clinical improvements observed.

In this study, the greater improvements observed in the younger adolescents likely reflect physiological differences, such as greater spinal flexibility, growth potential, and neuroplasticity (2, 19). Furthermore, younger participants may also benefit from higher parental involvement, which may enhance adherence and engagement with at-home programs (19). These findings reinforce the importance of early detection and timely conservative program before structural rigidity becomes a limiting factor.

Despite promising outcomes, several methodological limitations warrant caution. The single-arm design without a control group precludes causal attribution of the observed changes solely to the exercise intervention. Recruitment from a single geographic region may limit generalizability to broader populations or healthcare settings, and the absence of long-term follow-up prevents assessment of the durability of gains beyond the six-month intervention period. Future research should address these issues through multi-center randomized controlled trials, longer follow-up, and standardized reporting of adherence and intervention fidelity metrics. Other scoliosis-specific physiotherapeutic approaches—such as the SEAS method—were not implemented in the present study. Future comparative studies are warranted to evaluate whether Schroth–Pilates confers distinct advantages over SEAS or other structured exercise protocols in terms of radiographic and functional outcomes.

Taken together, the results indicate that online delivery of scoliosis-specific exercise programs can be both feasible and clinically relevant when supported by structured supervision and caregiver engagement. Future studies should (1) compare online, hybrid, and in-person modalities in randomized designs; (2) identify which program components (e.g., real-time supervision, asynchronous practice, wearable sensors, or video feedback) contribute most strongly to outcomes; and (3) extend follow-up to examine long-term sustainability.

## 5 Conclusion

This prospective cohort study provides observational evidence that a six-month, supervised Schroth-Pilates exercise program, delivered entirely online and in conjunction with brace treatment, can lead to meaningful improvements in both spinal curvature and trunk rotation in adolescents with IS. The program was feasible, well-accepted, and achieved high levels of adherence, with particularly notable benefits observed among younger adolescents aged 10–13 years. These findings underscore the importance of early, conservative, and structured program for scoliosis and demonstrate that virtual platforms can serve as effective channels for delivering complex rehabilitation protocols. Overall, the results highlight the potential of combined, home-based exercise modalities as part of comprehensive scoliosis care—especially in age-sensitive formats and contexts where in-person treatment may be limited. Future randomized controlled trials with longer follow-up periods are warranted to confirm these outcomes and further explore their long-term sustainability.

## Data Availability

The raw data supporting the conclusions of this article will be made available by the authors, without undue reservation.
