# Supplementary material for: Feasibility and age-related trends of a home-based online exercise program in adolescents with idiopathic scoliosis
Source: Front Pediatr. 2025 Dec 16;13:1695963. doi: 10.3389/fped.2025.1695963 (PMC12748210; doi:10.3389/fped.2025.1695963)
Supplement: Supplementary file 1 [file Table1.docx]

**STROBE Statement Checklist for Cohort Studies (Supplementary File S1)**

| Item No. | Recommendation | How Addressed / Location in Manuscript |
| --- | --- | --- |
| **Title and Abstract** |  |  |
| 1 | Indicate the study’s design with a commonly used term in the title or the abstract. | Title includes “prospective cohort study”; Abstract specifies “single-arm prospective cohort study”. |
| **Introduction** |  |  |
| 2 | Explain the scientific background and rationale for the investigation. | Introduction, paragraphs 1–3. Comprehensive overview of IS pathophysiology and treatment context. |
| 3 | State specific objectives, including any prespecified hypotheses. | Introduction, final paragraph. Objectives and age-related hypotheses described. |
| **Methods** |  |  |
| 4 | Present key elements of study design early in the paper. | Methods, first paragraph (“single-center prospective cohort design”). |
| 5 | Describe the setting, locations, and relevant dates. | Section 2.1 Participants and Study Design. Single-center, 6-month duration, recruitment period specified. |
| 6 | Describe eligibility criteria and the sources and methods of selection of participants. | Section 2.1 Participants – Inclusion/exclusion criteria listed. |
| 7 | Clearly define all outcomes, exposures, predictors, potential confounders, and effect modifiers. | Section 2.3 Measurements – Cobb angle, ATR, adherence, brace compliance defined. |
| 8 | Give diagnostic criteria, if applicable. | Section 2.1 and 2.3 – Diagnosis of IS confirmed by radiographic Cobb angle criteria (10°–45°). |
| 9 | For each variable, give sources of data and details of methods of assessment. | Section 2.3 Measurements – Describes radiographic measurement protocols, ATR measurement with scoliometer, and reliability testing. |
| 10 | Describe efforts to address potential sources of bias. | Section 2.5 Limitations – discusses lack of control group, single-center sampling, and blinded measurement for ATR and Cobb angle. |
| 11 | Explain how the study size was arrived at. | Section 2.1 – Sample size determined by available eligible participants over 6 months. |
| 12 | Explain how quantitative variables were handled in the analyses. | Section 2.4 Statistical Methods – continuous variables summarized as mean±SD; comparisons via t-tests and ANOVA. |
| 13 | Describe all statistical methods, including those used to control for confounding. | Section 2.4 Statistical Methods – independent t-tests, chi-square, mixed ANOVA, Bonferroni post-hoc adjustments. |
| **Results** |  |  |
| 14 | Report numbers of individuals at each stage of study (e.g., numbers potentially eligible, examined for eligibility, confirmed eligible, included, completed follow-up). | Section 3.1 Results and Figure 1 (Flowchart). |
| 15 | Give characteristics of study participants (e.g., demographic, clinical) and information on exposures and potential confounders. | Table 1 – Baseline characteristics; Section 3.1 Results. |
| 16 | Indicate number of participants with missing data for each variable of interest. | Section 3.1 Results – missing data <3%, addressed in text. |
| 17 | Report numbers of outcome events or summary measures over time. | Section 3.2 Results – improvements in Cobb angle and ATR over 6 months. |
| 18 | Summarize main results with unadjusted estimates and, if applicable, adjusted estimates and their precision (e.g., 95% CI). | Section 3.2 and Table 2 – F-values, p-values, partial η² provided. |
| **Discussion** |  |  |
| 19 | Discuss limitations of the study, taking into account potential sources of bias or imprecision. | Discussion, final paragraphs. Addresses lack of control group, regional recruitment, and short-term follow-up. |
| 20 | Give a cautious overall interpretation of results considering objectives, limitations, multiplicity of analyses, and results from similar studies. | Discussion, paragraphs 2–6. Critical comparison with prior Schroth, Pilates, and tele-rehab studies. |
| 21 | Discuss the generalisability (external validity) of the study results. | Discussion, final paragraph – applicability to remote/low-resource settings. |
| **Other Information** |  |  |
| 22 | Give the source of funding and the role of the funders. | Section 6 Acknowledgements/Funding – declares institutional support, no commercial role. |

**Note:** This checklist follows the STROBE (Strengthening the Reporting of Observational Studies in Epidemiology) guidelines for cohort studies. All corresponding sections and page numbers refer to the revised manuscript titled *“Feasibility and Age-Related Trends of a Home-Based Online Exercise Program in Adolescents with Idiopathic Scoliosis.”*
